# Whole Genome Sequencing Shows Genetic Diversity, as Well as Clonal Complex and Gene Polymorphisms Associated with Fluconazole Non-Susceptible Isolates of *Candida tropicalis*

**DOI:** 10.3390/jof8090896

**Published:** 2022-08-23

**Authors:** Caitlin Keighley, Mailie Gall, Sebastiaan J. van Hal, Catriona L. Halliday, Louis Yi Ann Chai, Kean Lee Chew, Chayanika Biswas, Monica A. Slavin, Wieland Meyer, Vitali Sintchenko, Sharon C. A. Chen

**Affiliations:** 1 Centre for Infectious Diseases and Microbiology Laboratory Services, Institute of Clinical Pathology and Medical Research, New South Wales Health Pathology, Sydney, NSW 2145, Australia; 2 Centre for Infectious Diseases and Microbiology, Sydney Institute for Infectious Diseases, The University of Sydney, Westmead Hospital, Sydney, NSW 2145, Australia; 3 Department of Infectious Diseases and Microbiology, New South Wales Health Pathology, Royal Prince Alfred Hospital, Sydney, NSW 2050, Australia; 4 Division of Infectious Diseases, Department of Medicine, National University Health System, Singapore 119228, Singapore; 5 Yong Loo Lin School of Medicine, National University of Singapore, Singapore 117597, Singapore; 6 Department of Laboratory Medicine, National University Health System, Singapore 119074, Singapore; 7 Department of Infectious Diseases, National Centre for Infections in Cancer, Peter MacCallum Cancer Centre, Melbourne, VIC 3000, Australia; 8 Molecular Mycology Research Laboratory, Center for Infectious Diseases and Microbiology, Westmead Institute for Medical Research, Westmead, NSW 2145, Australia; 9 Research and Education Network, Western Sydney Local Health District, Westmead Hospital, Westmead, NSW 2145, Australia; 10 Curtin Medical School, Curtin University, Bentley, WA 6102, Australia

**Keywords:** *Candida tropicalis*, fungal infections, antifungal drug resistance, whole genome sequencing, genetic variation, azoles, echinocandins, MLST

## Abstract

Resistance to azoles in *Candida tropicalis* is increasing and may be mediated by genetic characteristics. Using whole genome sequencing (WGS), we examined the genetic diversity of 82 bloodstream *C. tropicalis* isolates from two countries and one ATCC strain in a global context. Multilocus sequence typing (MLST) and single nucleotide polymorphism (SNP)-based phylogenies were generated. Minimum inhibitory concentrations (MIC) for antifungal agents were determined using Sensititre YeastOne YO10. Eleven (13.2%) isolates were fluconazole-resistant and 17 (20.5%) were classified as fluconazole-non susceptible (FNS). Together with four Canadian isolates, the genomes of 12 fluconazole-resistant (18 FNS) and 69 fluconazole-susceptible strains were examined for gene mutations associated with drug resistance. Fluconazole-resistant isolates contained a mean of 56 non-synonymous SNPs per isolate in contrast to 36 SNPs in fluconazole-susceptible isolates (interquartile range [IQR] 46–59 vs. 31–48 respectively; *p* < 0.001). Ten of 18 FNS isolates contained missense *ERG11* mutations (amino acid substitutions S154F, Y132F, Y257H). Two echinocandin-non susceptible isolates had homozygous *FKS1* mutations (S30P). MLST identified high genetic diversity with 61 diploid sequence types (DSTs), including 53 new DSTs. All four isolates in DST 773 were fluconazole-resistant within clonal complex 2. WGS showed high genetic variation in invasive *C. tropicalis*; azole resistance was distributed across different lineages but with DST 773 associated with in vitro fluconazole resistance.

## 1. Introduction

*Candida tropicalis* is an important cause of invasive candidiasis with a 30-day mortality as high as 40–50% [1,2]. Although it typically ranks third or fourth as a cause of candidemia [3,4,5,6], it is the most frequent causative species in some regions of the Asia Pacific [7,8,9] and the second most common species in Latin America [10]. *C. tropicalis* is considered susceptible to many antifungal agents, however, resistance to the azoles has been increasing reported [4,11,12,13]. In Australia, surveillance of *C. tropicalis* bloodstream isolates demonstrated a rise in fluconazole resistance from <2% in the mid-2000s to 16.7% a decade later [4,14] prompting the need to better understand drug resistance in this species.

The mechanisms of resistance to azole antifungals in *C. tropicalis* are likely to be multifactorial as it is with other *Candida* species [15,16]. Certain mutations in the azole target lanosterol 14-alpha-demethylase (Erg11p) encoded by the *ERG11* gene (e.g., those leading to the Y132F amino acid substitution) have been linked to high-level azole resistance [17,18,19,20,21]. *ERG11* overexpression may also play a role in drug resistance [22] as does increased expression of the genes, *CDR1* and *MDR1*, which encode for drug efflux transporters [23,24]; the latter are under the control of the genes *TAC1* and *MMR1*, which encode for transcription regulators. Of note, changes resulting in increased copy number of *ERG11* and *TAC1* as well as loss of heterozygosity (LOH) in *MMR1*, *ERG11* and *TAC1*, have also been associated with resistance to fluconazole, in keeping with *C. tropicalis* being a diploid organism [18,21,25,26,27,28,29].

Genotyping approaches offer novel insights into drug resistance that supplement conventional assessment of drug susceptibility by the minimum inhibitory concentration (MIC) phenotype. Using multilocus sequence typing (MLST), extensive genetic diversity has been reported with multiple *C. tropicalis* diploid sequence types (DSTs) described, which are grouped within distinct clonal complexes [30,31,32,33]. Whereas some data suggest no association between DST and susceptibility to the azoles, in particular, fluconazole [30,31,32], others have linked a particular DST, e.g., DST225, or clonal complexes, e.g., clonal complex 2 (CC2), to isolates considered to be phenotypically fluconazole non-susceptible (FNS) [17,33,34,35,36].

Recently, whole genome sequencing (WGS) approaches have been applied to study drug resistance in *C. tropicalis* [29,37] and its phylogenetic characterization [38]. O’Brien et al. demonstrated varying degrees of heterozygosity across the genomes of 77 clinical and environmental *C. tropicalis* isolates with no apparent association between susceptibility to fluconazole and genotypes. Though MIC values were not reported, only three isolates exhibited decreased susceptibility to fluconazole [38]. In a small Canadian study of a single pair of *C. tropicalis* isolates, an increased copy number of *ERG11* was documented after fluconazole therapy in association with fluconazole resistance (MIC 32 mg/L) [29]. However, these studies using WGS to assess genomic features conferring azole resistance have included either small numbers of isolates (<5) and/or few susceptible strains for comparison.

We have previously demonstrated the value of WGS in investigating genetic variation and drug resistance in *Candida glabrata* and *Candida auris* [39,40]. Here, we aimed to utilize WGS to firstly study the genetic diversity of Australian *C. tropicalis* isolates in a global context and to compare azole non-susceptible isolates from two countries (Australia and Singapore), with those that were azole-susceptible for presence of *ERG11* mutations, and mutations in other genes implicated in azole resistance. We also examined the copy number of *ERG11* and *TAC1* as well as LOH in *ERG11*, *TAC1* and *MMR1*.

## 2. Materials and Methods

### 2.1. Isolates and Susceptibility Testing

A total of 83 *C. tropicalis* isolates were studied. These comprised 82 clinical bloodstream isolates: 50 were from two national Australian surveys [4], 22 were additional isolates from Australia spanning 2010–2021 and 10 were from Singapore. The reference strain of *C. tropicalis* ATCC 13803 was also included. All isolates were obtained from culture collections at the Clinical Mycology Laboratory, Westmead Hospital, the Molecular Mycology Research Laboratory, Westmead Institute for Medical Research, Sydney, Australia, and from the National University Hospital, Singapore. Details of the isolates and associated metadata are given in Table S1.

In addition, genome sequences for 73 previously-reported isolates with limited metadata were included for comparison and to provide context; these included 4 isolates from Canada with MIC data [29] included into the drug resistance and phylogenetic analyses. Further, 69 isolates from the USA, Ireland, India, Germany, Spain, Columbia and the Netherlands but without MIC data [38] were incorporated into the phylogenetic analysis only (Table S2). These are referred to by the accession number assigned for the sequence data; isolate names may be obtained from the relevant publications [29,38].

For the study, identification of all isolates was confirmed by matrix-assisted laser desorption ionization-time of flight technique (MALDI-TOF MS; Biotyper database v3.1, Bruker Daltonics, Billerica, MA, USA) supplemented by ITS sequencing [4]. Susceptibility testing was determined using Sensititre YeastOne YO10 methodology (TREK Diagnostics, Cleveland, OH, USA) in accordance with the manufacturer’s instructions. MICs were determined after 24 h incubation at 35 °C and interpreted according to species-specific Clinical and Laboratory Standards Institute (CLSI) breakpoints [41,42]. *Candida krusei* ATCC 6258 and *Candida parapsilosis* ATCC 22019 were used as quality control strains. Fluconazole susceptibility was defined as an MIC of ≤2 mg/L: susceptible-dose dependent as MIC of 4 mg/L, whereas isolates with MICs of ≥8 mg/L were classed as resistant. Fluconazole non-susceptible (FNS) isolates were defined as those with MIC of ≥4 mg/L.

For micafungin and anidulafungin, isolates were classified as susceptible if the MIC was below 0.5 mg/L, intermediate at 0.5 mg/L and resistant at >0.5 mg/L. Amphotericin B non-wild type (non-WT) strains were those with an amphotericin B MIC of >2 mg/L. Interpretation of MICs for other antifungal agents were in accordance with CLSI guidelines [41,42].

### 2.2. DNA Extraction, PCR Amplification and Sequencing

All isolates were grown on Sabouraud’s dextrose agar (SDA) incubated at 35 °C for 48 h and checked for purity prior to experiments. Total genomic DNA extraction was performed using the The MasterPureTM Yeast DNA kit (Illumina, San Diego, CA, USA). The DNA concentration was then quantified using the Quant-iT PicoGreen dsDNA Assay kit (Life Technologies, Carlsbad, CA, USA). Genomic libraries were constructed using the Nextera XT DNA prep kit (Illumina, San Diego, CA, USA). Tagmentation, PCR amplification and clean up, library normalisation and pooling and sequencing were performed on the NextSeq 500 or MiSeq 500 platforms (Illumina), carried out with 2× 150-bp paired-end chemistry. Isolates were sequenced to a coverage of 41×–177×. The median read coverage was 115× (interquartile range 94×–132×).

### 2.3. Whole Genome Sequencing Analysis

Raw paired fastq files were trimmed using Trimmomatic v0.36 with default parameters (Bolger et al., 2014). The quality of the trimmed fastq files was evaluated using FastQC v0.11.3. Minimum read coverage of 40× after trimming was accepted for isolates and downloaded sequence data. Hybrid, highly heterozygous genomes as described by O’Brien et al. were excluded [38]. Possibility for laboratory cross-contamination was checked using Centrifuge v0.1.4-beta, with a database employed that is inclusive of prokaryotes, viruses and *Homo sapiens*.

Sequencing data were mapped to the reference genome *C. tropicalis* MYA-3404 (www.candidagenome.org/download/sequence/ accessed on 10 February 2021) assembly ASM633v3 (Accession GCF_000006335.3) to ensure consistency with annotations described in the literature. A more recent genome assembly based on PacBio sequencing of the same reference strain *C. tropicalis* MYA-3404 was employed to anchor analysis of large structural changes (Accession GCA_013177555.1). Reads were mapped to the reference using the Burrow—Wheeler alignment tool (BWA) v0.7.17-r1188 with default settings. Bam files were sorted and indexed using SAMtools v1.6 and duplicate reads marked using Picard MarkDuplicates v2-20-6.

Single nucleotide polymorphisms (SNPs) were predicted using FreeBayes (min alt 0.3, min depth 10) specifying a ploidy of 2. The VCF files were normalised using VCFlib v1.0.0. followed by annotation using SNPeff v4.3t [43]. All detected heterozygous sites were collapsed and represented by IUPAC ambiguity codes using the -I option within bcftools v1.9 to generate consensus sequences following masking of low coverage sites (depth < 10). The consensus sequence was used for both the MLST and SNP-based phylogenies (see sections below). The genomic data are available under the BioProject number PRJNA865384.

#### 2.3.1. MLST

MLST was determined using the consensus sequences. For each isolate, genomic regions spanning the known six housekeeping DST loci: *ICL1*, *MDR1*, *SAPT2*, *SAPt4*, *XYR1* and *ZWF1a* were extracted from the full genomic consensus sequence [44]. To determine the appropriate genomic region for each DST locus, a representative allele for each locus was mapped to the reference genome assembly ASM633v3 (Accession GCF_000006335.3). The positions on the reference for each locus were then extracted using bedtools. Each allele was run through a custom Python script which compared the consensus sequence to the public *C. tropicalis* MLST database (http://pubmlst.org/organisms/candida-tropicalis, accessed on 15 November 2021). The custom script incorporated Blast version 2.9.0+ to first create a database of DST alleles. Pairwise comparisons for matches ranked with highest similarity were performed to confirm exact allelic matches. Allelic profiles and DSTs were compared to those downloaded from the public MLST database in order to determine whether the combination of alleles represented a novel DST. The assignments are detailed in Table S1. A multifasta file containing alleles for the MLST sequences is also available as a Supplementary file. Clonal complexes were assigned using Phyloviz 2.0 with the goeBURST algorithm.

#### 2.3.2. Phylogenetic Analysis

The genetic relationships amongst the *C. tropicalis* isolates based on the MLST loci was constructed using GrapeTree version 1.5.0 [45]. A minimum spanning tree was generated based on the concatenated nucleotide sequences of each DST loci, which were then combined into a multifasta alignment containing all isolates. The MSTreeV2 algorithm was implemented using GrapeTree with all other parameters default. The network was visualised using the GrapeTree web-visualisation tool (https://achtman-lab.github.io/GrapeTree/MSTree_holder.html accessed on 20 July 2022).

The SNP-based phylogenetic relationships between isolates were examined by comparing whole genome consensus sequences. Low coverage (depth < 10) positions were masked as ‘N’ in the alignment. The genetic relationship was represented by a maximum-likelihood tree using IQ-TREE version 1.6.7 with 1000 bootstrap replicates and a GTR+GAMMA model. Clusterpicker version v1.2.5 was used to place samples into clusters using a bootstrap cutoff of 99% and a genetic distance of 0.003.

#### 2.3.3. Assessment of Antifungal Drug Resistance Markers

The list of genes known to be involved in azole or echinocandin resistance in *Candida* species was collated by searching the NCBI, PubMed and Embase literature in English for the key words: “fluconazole”, “azole”, “echinocandin”, “resistance”, “*Candida tropicalis*” and “*Candida* species” up to 30 July 2021. The database included in MARDy was also accessed (http://mardy.net/ accessed on 30 July 2021). Where genes had been described using reference coordinates different from the reference used in this study, the datasets were harmonized by mapping the published fasta sequences of interest to ASM633v3 using bwa v0.7, followed by bedtools v2.29.2, used to determine the appropriate coordinates on the reference assembly. A curated list of selected resistant genes and their coordinates is provided in Supplementary Table S3. These genes were further examined for the presence of mutations in the genomes of both azole non-susceptible (inclusive of azole resistant isolates), and azole susceptible isolates.

The large numbers of azole susceptible isolates studied herein (see Results) provided an opportunity to create a catalogue of gene variants potentially associated with in vitro resistance to assist with identifying novel candidate gene variants. The VCF files generated by Freebayes were filtered in R v3.5.2 to identify coordinates and amino acid substitutions, which matched the coordinates in the curated gene set. Only non-synonymous mutations were considered. To generate a list of potential variants associated with resistance, the VCF files were then filtered to exclude SNPs which were present in at least one phenotypically susceptible isolate. Candidate variants were manually curated by inspecting the bam files in IGV v2.6.3. To compare the number of mutations in candidate genes, a Mann–Whitney test was used.

We screened our isolates for copy number variants (CNVs) across the *ERG11* gene [21,25,28,29]. As part of CNVnator [45], reads were first mapped to the reference genome Assembly2020, because it has been resolved at the chromosomal level, which would ensure maximum continuity of coverage across the genome, as artificial breaks introduced by contigs might potentially affect our ability to detect CNVs (reference ASM633v3 is comprised of 24 contigs). We set a minimum threshold of a copy number change to be considered as candidate at 1.5×. Candidate CNVs were confirmed as true with manual curation through visualization of the bam files in IGV version 2.4.10.

Finally, we searched for LOH events across *ERG11*, *TAC1* and *MMR1* [27] using a custom R script. This script calculates the frequency of heterozygosity/homozygosity across the genome, using vcf files generated by FreeBayes, and normalised with VCFlib. For each variant position, frequencies were calculated across a 10,000 window, with a region considered as LOH if the rate of heterozygosity consistently dropped below 0.1%. Plots representing potential LOH regions were generated using the ggplot2 library in R. Areas, suggesting increased copy number change or LOH were manually curated by visualization in IGV.

## 3. Results

### 3.1. Phenotypic Susceptibility to Antifungal Agents

Susceptibility testing results for the Australian, Singapore and ATCC study isolates (*n* = 83) are summarised in Table 1 (please also see Table S1 for further details).

Of 83 isolates, 17 (20.4%) were FNS (MICs ranging from 4 to >256 mg/L), 11 (13.2%) of these were resistant to fluconazole and 66 (79.5%) were fluconazole-susceptible (Table 1 and Table S1). With the addition of four isolates described by McTaggart et al., including one isolate, designated as SRR11235418 that was fluconazole resistant (fluconazole MIC 32 mg/L; Table S2) [29], the total number of isolates assigned to each of these categories for genomic analysis was then 18, 12 and 69, respectively. Of the 18 FNS isolates, 16 (89%) were also non-susceptible to voriconazole (MIC range 0.25 to >8 mg/L) (Table 2). Azole non-susceptible isolates spanned over 20 years with no epidemiological clusters.

Echinocandin resistance (micafungin MIC 4 mg/L, anidulafungin MIC 0.5 mg/L) was present in one isolate, strain 19-008-0018 from Singapore and in a further isolate, designation SRR11235416 from Canada [29] (MIC (mg/L): micafungin 2, anidulafungin 1) (Table 2 and Table S2). None of the study isolates had non-WT MICs to amphotericin B or flucytosine.

### 3.2. Multilocus Sequence Typing (MLST)

For 83 isolates, 61 DSTs were evident (Table S1). The *MDR1* and *SAPT4* loci showed the greatest degree of polymorphism (24 unique alleles), followed by *XYR1* (17 unique alleles), *SAPT2* (12 unique alleles), with *ICL1* showing the lowest polymorphism (8 unique alleles). Eight DSTs (145 [5 isolates], 168 [1 isolate], 275 [3 isolates], 335 [1 isolate], 678 [4 isolates], 773 [4 isolates], 1091 [1 isolate] and 1211 [1 isolate]) have been previously reported matching DSTs in the public MLST database. Thus, there were 53 novel DSTs. Twelve of the 61 DSTs were present in at least two isolates with the remaining 49 assigned only to a single isolate. 

**Figure 1:**
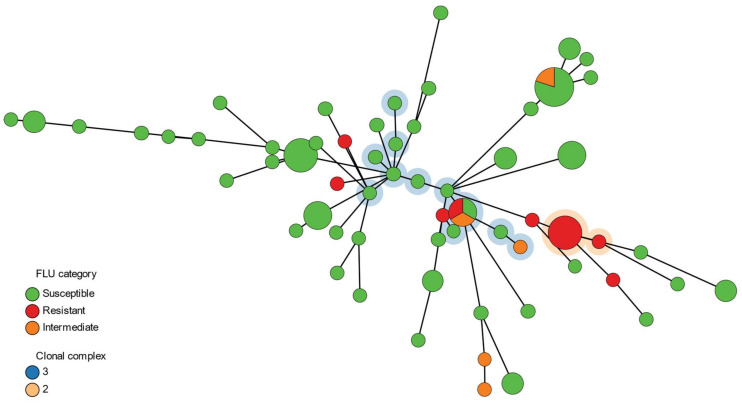
Minimum spanning tree illustrating the relationships between the 83 study isolates, generated from the concatenated DST nucleotide sequence data. Each node represents a unique DST, with the size of the node proportional to the number of isolates represented by that node/DST. The edges represent the allelic distance between nodes. Clonal complexes of interest 2 and 3 are highlighted. FLU, fluconazole.

DST 773 comprised three isolates from Singapore (19-008-0007, 19-008-0008 and 19-008-0017) and one from Australia (19-5326-037-0003), all with fluconazole MICs of 256 mg/L. Along with one other Singapore isolate with high level fluconazole resistance (19-008-0016, MIC > 256mg/L) but which was assigned to DST 1299, these isolates belonged to clonal complex 2 (CC2) (Figure 1). There were no other isolates within this clonal complex. Clonal complex 3 comprised four Australian FNS isolates with MIC ranging from 4 to 64 mg/L, as well as 10 fluconazole susceptible isolates (Figure 1). DST 145 included one fluconazole susceptible dose-dependent isolate and four fluconazole-susceptible isolates from different hospitals in the Australian jurisdictions of New South Wales (NSW) and Queensland. DST 678 comprised only fluconazole-susceptible isolates collected from different hospitals in NSW.

### 3.3. Phylogenetic Analysis

**Figure 2:**
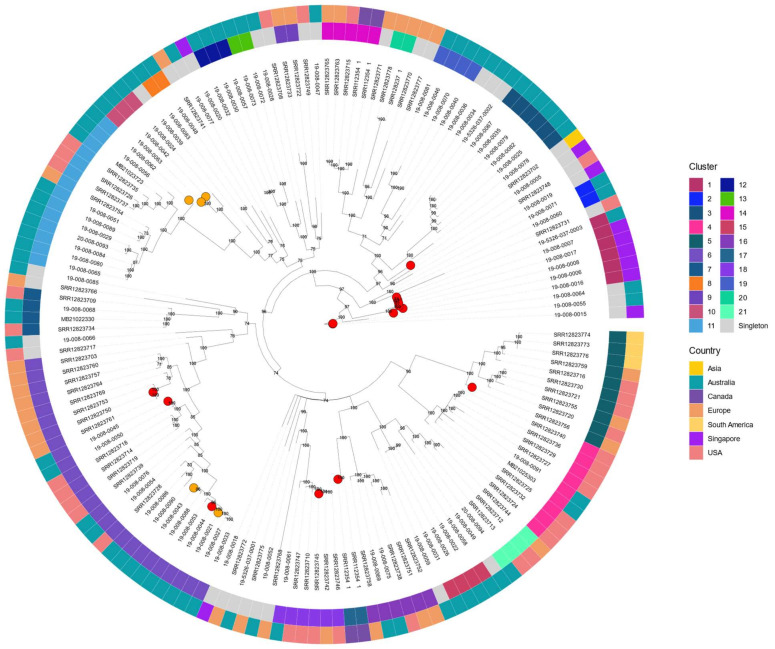
Maximum-likelihood tree (midpoint root) of 156 isolates based on whole genome sequences and a GTR+GAMMA model. Bootstrap support values are shown. Susceptibility to fluconazole for FNS isolates is denoted by the filled circles at the tree tips with red colour indicating resistant and orange indicating susceptible, dose dependent. Inner ring denotes cluster designation by number. Outer ring denotes country or continent of origin.

Phylogenetic analysis demonstrated that the *C. tropicalis* study isolates were genetically diverse. Other than one cluster (consisting of three Singaporean and an Australian isolate that were FNS), Australian and Singaporean isolates were dispersed across the phylogeny with international isolates from other geographical locations (Figure 2 and Table S1). Clusterpicker classified sequences into 20 clusters ranging in cluster size from 2 to 18 (Figure 2). Cluster 1 comprised six isolates that were all fluconazole-resistant; this included the five isolates that belonged to CC2 above and an additional isolate from Singapore (19-008-0006, fluconazole MIC of >256 mg/L, DST1339) (Figure 2, Table S1). All of these isolates had both the S154F and Y132F amino acid substitutions in *ERG11* occurring in the homozygous form (Table 2). FNS isolates were also found within clusters 6, 10, 11 and 21 but along with fluconazole-susceptible isolates. There were no fluconazole-susceptible isolates within cluster 1 (Figure 2).

### 3.4. Gene Mutations and Antifungal Resistance

The genomes of 18 fluconazole non-susceptible (FNS), two echinocandin non-susceptible as well as 67 drug susceptible *C. tropicalis* isolates were examined for mutations in the following genes: *ERG11*, *ERG6*, *TPO3*, *ERG3*, *FLU1*, *Ndt80*, *UPC2*, *STB5*, *TAC1*, *MDR1*, *MMR1*, *SNQ2*, *YOR1*, *CDR11*, *CDR1* (for azole resistance), *RHO1*, *FKS1*, *FKS2* and *FKS3* (echinocandin resistance) (Table S3). Compared with the corresponding genome sequences of *C. tropicalis* MYA-3404, we identified 403 distinct non-synonymous polymorphisms (see Tables S4 and S5 for details).

#### 3.4.1. Azole Resistance

There were four non-synonymous polymorphisms in *ERG11*, 18 in *TAC1*, 28 in *MMR1*, 115 in *CDR1*, 12 in *UPC2*, 4 in *ERG6*, 3 in *TPO3*, 7 in *ERG3*, 8 in *FLU1*, 25 in *MDR1*, 6 in Ndt80, 13 in *STB5*, 27 in *SNQ2*, 31 in *YOR1* and 34 in the *CDR11* genes-335 in total (see also below).

For the 12 fluconazole-resistant isolates, there was a mean of 56 non-synonymous SNPs in the listed genes of interest (interquartile range, IQR 46–59) compared with a mean of 36 (IQR 31–48) for fluconazole-susceptible isolates (*p* < 0.001). Fluconazole susceptible dose-dependent isolates (*n* = 6), however, had fewer SNPs (mean 30, IQR 24–33) (*p* < 0.05). A low frequency (<1.5%, corresponding to presence of the mutation in only one strain) was observed for 111 (33%) mutations. The details of both heterozygous and homozygous gene mutations are shown in Table S4.

**Figure 3:**
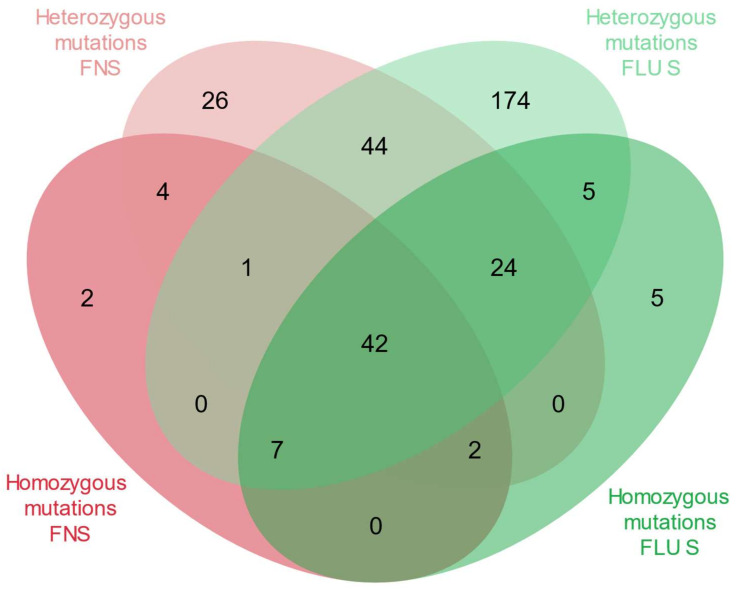
Distribution of counts of amino-acid substitutions in genes involved in azole resistance grouped by fluconazole susceptibility and by presence of heterozygous and/or homozygous mutations. FNS, fluconazole non-susceptible; FLU, fluconazole; S, susceptible.

From the catalogue of gene variants established in the present study (see Methods), of the 335 non-synonymous SNPs in genes associated with azole resistance, seven (2%) were found in the homozygous state only, 244 (73%) were found in the heterozygous state only and 85 (25%) were noted in both heterozygous and homozygous states, as shown in Figure 3.

We further identified amino acid substitutions in *ERG11* and other genes as shown in Table 3. Variants present only in FNS isolates were documented in 18 isolates, 10 demonstrated recognised missense resistance-conferring amino acid substitutions in *ERG11*, leading to the substitutions S154F, Y132F, Y257H (Table 2, Table 3 and Table S4) associated with fluconazole MICs ranging from 8 to over 256 mg/L. These *ERG11* variants were absent in fluconazole-susceptible isolates.

The genomes of eight isolates with MICs between 4–32 mg/L had no mutations in the *ERG11* gene (Table 2). Four of these isolates (19-008-0042, 19-008-0044, 19-008-0062 and 19-008-0063) had one or more amino acid substitutions in the genes investigated for azole resistance (see methods) not present in susceptible isolates, but only one strain 19-008-0042 had a mutation detected in the homozygous form in the *CDR11* gene, leading to substitution D779Y (Table S4).

None of the Australian or Singapore isolates had a CNV in *ERG11* [20]. Loss of heterozygosity in *MMR1* was present in 6 FNS and 11 fluconazole-susceptible isolates (Figure S1) as was loss of heterozygosity in *ERG11/TAC1* (Table S6). One FNS isolate (MB21022330, fluconazole MIC 4 mg/L) had no candidate amino acid substitutions, CNV or LOH identified.

#### 3.4.2. Echinocandin Resistance

There were four non-synonymous polymorphisms in *C. tropicalis FKS1*, 28 in the *FKS2*, 36 in the *FKS3* genes but none in *RHO1* (Table S5). Of the 68 variants, seven (10%) were found in the homozygous state only, 49 (72%) in the heterozygous state only with 12 (18%) noted in both heterozygous and homozygous states. The two echinocandin non-susceptible isolates had a homozygous mutation in the *FKS1* gene (CTRG_04661) leading to amino acid substitution S30P. This had been previously noted for isolate SRR11235416 [29]. The S30P mutation was absent in echinocandin-susceptible isolates.

## 4. Discussion

This study extends the understanding of the genomic diversity of *C. tropicalis* in the context of the antifungal susceptibility patterns, which is essential to managing infections caused by this pathogen. Our findings have demonstrated a high degree of genetic diversity within this species using WGS to study the genomes of a large number of clinical *C. tropicalis* isolates. We also determined the presence of gene mutations associated with clinically relevant drug resistance in the context of the drug susceptibility phenotype. Main findings were that fluconazole resistance, and non-susceptibility (FNS) were associated with isolates assigned to CC2 and to the MLST type, DST 773. The importance of S154F, Y132F and Y257H amino-acid substitutions in conferring azole resistance, as in multiple *Candida* species, was affirmed [29,37,46].

The frequency of fluconazole resistance was relatively high at 20.5% among isolates in this study compared with 16.7% in a previous Australian study [4] and other studies globally (3.1–23.1%) [34,46,47]. Because of potential sampling bias, however, this cannot be extrapolated to determining trends. The Australian study isolates were supplemented by azole-resistant Singaporean isolates to enable a larger number of azole-resistant isolates for genetic analysis. Importantly, the relatively large number of fluconazole-susceptible isolates herein also enabled the establishment of a catalogue of gene mutations or variants which are potentially associated with in vitro resistance, which can assist with identifying novel gene variants that confer resistance.

Of note, we found fluconazole-resistance, as defined by current MIC breakpoints, to be associated with certain *C. tropicalis* clonal complexes, namely CC2, and clusters, namely cluster 1 (see Results: phylogeny), building on previous reports [11,19,36]. Australian and Singapore isolates belonging to CC2, included four isolates assigned to DST 773 and all were fluconazole-resistant; further, they carried mutations in the *ERG11* gene [11,17,19,36]. Conversely, no fluconazole susceptible isolates were assigned to CC2 or DTS 773. Likewise, CC 2 has been reported to contain isolates that were resistant to fluconazole in a recent report from China, though assessment for *ERG11* mutations was not performed [36]. However, other studies have demonstrated fluconazole-susceptible isolates within CC2 [34,35]. There are data to support the development of CC2 azole-resistant *C. tropicalis* with use of agricultural azoles, the practice of which varies with geography [13,48]. Of interest, there were no DST 225, 376, 505–7, 525 or 546, also previously associated with FNS isolates [17,33,34,35,36]. The absence of these DST types amongst our FNS isolates is likely related to geographical differences in distribution of genotypes. Indeed, only eight DSTs described in this study matched previously assigned DSTs largely reported from Taiwan, China and Japan [17,33,34,35,36]. The large number of novel DSTs documented in this study builds on the public MLST database for future data sharing. As expected, phylogenetic analysis confirmed the genetic diversity of the study isolates by MLST, further supporting the hypothesis that *C. tropicalis* isolates, at least those from the bloodstream, have arisen from disparate environments. In contrast with previous reports, we found the clonal complex CC3 to contain both fluconazole susceptible and non-susceptible isolates [17]. Whether the association between fluconazole resistance/FNS and CC2, DST 773 and cluster 1 is supported will require further studies.

Importantly, our findings support an association of phenotypic fluconazole resistance with specific *ERG11* mutations. Seven isolates with two mutations in the *ERG11* gene (S154F and Y132F) in the homozygous form had the highest MICs to fluconazole (128–>256 mg/L, Table 2). Such substitutions have been proven to confer resistance when a plasmid with these resistance mutations was transferred from *C. tropicalis* to *S. cerevisiae* [46], and have explained fluconazole resistance in a number of studies [19,21,37,46,47,49,50]. Further, these mutations were absent in azole susceptible isolates. Consistent with our results, the occurrence of multiple homozygous mutations in *ERG11* was associated with a more pronounced increase in MIC to azoles in *C. tropicalis* as it has for *C. albicans* [17,21]. Conversely, we noted that isolates with a single homozygous mutation in *ERG11*, or with two mutations in the heterozygous form had a lower MICs to fluconazole (range 8–64 mg/L) (Table 2). Amino acid substitutions in *ERG11* were the most common explanation for fluconazole non-susceptibility, with a possible explanation discovered in 94% of sequenced genomes.

Overall, fluconazole-resistant isolates had a greater number of non-synonymous SNPs in genes involved in azole resistance per isolate compared with fluconazole-susceptible isolates, suggesting selection pressure and thus an increased mutation rate may lead to drug resistance. The same, however, was not true for fluconazole susceptible, dose-dependent isolates. This discordance may simply reflect the small number of isolates (*n* = 6) in this group. It may also represent a bias due to the resistant isolates that were included, with many of them clustering together, though the finding is consistent with what has been recently described in *C. albicans* [51]. As with *C. albicans*, azole resistance appears to be multifactorial in *C. tropicalis* [15,16]. Although we did observe multiple SNPs in the drug efflux transporter genes and those that encode for transcription regulators, e.g., *CDR1*, *MDR1*, *CDR11*, *TAC1* and *MMR1*, the presence of SNPs *per se* does not confer resistance and data from up-regulation or gene expression studies are required [52]. Of FNS isolates not explained by the *ERG11* mutations, only one (fluconazole MIC 4 mg/L) had a homozygous mutation in the *CDR11* gene that was not present in susceptible isolates. However, it seems unlikely that this would have accounted for the MIC above 2 mg/L. A further isolate 19-005-0044 with a fluconazole MIC of 32 mg/L, LOH in *MMR1* was identified alongside a heterozygous mutation in *SNQ2* and *TPO3.* LOH events can provide a rapid adaptive advantage inducing phenotypic shifts by unmasking recessive variants that might confer a fitness benefit [25,26,27]. Whether these mutations explain the phenotype remains unconfirmed. Additionally, the MIC of 4 mg/L in 6 of the 8 FNS isolates without *ERG11* mutations may be within the error of MIC measurement and reflect a true MIC of only 2 mg/L. Nonetheless, it is notable that none of the isolates without an *ERG11* mutation had a fluconazole MIC above 32 mg/L.

Copy number variants (CNVs) have been shown to be inducible in response to stress and a mechanism whereby *C. tropicalis* may acquire resistance [21,25,28,29]. Our findings confirmed the increased copy number including *ERG11* and *TAC1* in a previously described azole-resistant isolate following a period of antifungal therapy [29] and where the structural feature was absent in the paired azole-susceptible isolate cultured prior to exposure to antifungals. Hence, structural changes are more likely to occur after azole therapy [18,53]. In our study, increased copy numbers in *ERG11* and *TAC1* were not found; all study isolates were the ‘first’ isolate recovered from the patient. That CNVnator was able to detect the previously reported finding confirms the usefulness of this tool in assessing for duplication events. Finally, in seven FNS isolates, LOH of *ERG11* or *MMR1* were demonstrated (see above). However, the presence of these features also in fluconazole-susceptible isolates likely indicates either that this is insufficient to cause resistance, or that other features were present that counteract their effect.

The present study found only one isolate that was echinocandin-resistant and assessed sequence data for another known echinocandin-resistant isolate. Both had a homozygous S30P mutation, which is aligned with the S645P amino acid substitution in the hotspot 1 region in the homologous gene of *C. albicans*, as previously noted [29].

Some limitations in our study have to be acknowledged. We did not have access to clinical information. Detail regarding treatment outcomes would have been informative for assessing the clinical impact of WGS-enabled markers of antifungal drug resistance. Further, assessment of transcription of genes and gene expression studies was beyond the scope of this study to determine the multifactorial natures of drug resistance. Our set of isolates also had a potential selection bias and included only two isolates with resistance to echinocandins. However, strengths of the study include a large number of unrelated clinical isolates which provided essential data on background diversity in *C. tropicalis*. It allowed for detection of markers of resistance in disparate lineages, providing evidence of the same mutation arising independent of lineage. It also assessed for CNV and LOH in fluconazole-susceptible compared with FNS isolates, with use of the chromosomal assembly of the *C. tropicalis* reference [54]. Although paired isolates studies allow a subtraction analysis of features in susceptible/non-susceptible isolates, they are limited in that they only detect mutations that are induced by antifungal therapy, rather than those that occur spontaneously. The study was also limited to describing the genetic associations (if any) for fluconazole susceptibility and was not designed for extrapolation of results to the other azoles.

Finally, there is no comprehensive database for drug resistance in fungi as exists for medically relevant bacteria and a number of challenges in interpreting genomic markers of antifungal resistance still remain. For example, the amino acid substitution positions depend on the reference genome used for comparative analysis, with the example of S30P in the *FKS1* gene in our dataset. There is yet to be a standard approach to analysis of structural variation in diploid organisms.

## 5. Conclusions

The present study assembled a large collection of fully sequenced genomes of invasive isolates of *C. tropicalis*. Whole genome sequencing enabled high-resolution analysis of genomic diversity and markers potentially associated with clinically relevant resistance to antifungal agents in *C. tropicalis*.

Significant genetic diversity across Australia and to a lesser extent, Singapore, was identified with all major lineages represented. Notably, the findings support the association of CC2 and DST 773 with in vitro fluconazole resistance. This study paves the way for cataloguing genomic markers of clinically relevant resistance in Candida species.

## Figures and Tables

**Table 1:** In vitro susceptibilities of 83 *Candida tropicalis* isolates to nine antifungal agents.

				MICs (mg/L)					
	FLU	VOR	POS	ITR	AMP	5FC	CAS	MCF	ANF
**MIC range**	0.5–>256	0.015–>8	0.03–2	0.06–>16	<0.12–1	<0.06–1	0.008–4	<0.008–4	<0.015–0.5
**MIC90**	32	2	0.5	0.5	1	0.12	0.25	0.03	0.12
**GM MIC**	2.36	0.18	0.19	0.28	0.78	0.08	0.06	0.02	0.06
**S or WT *n* (%)**	66 (79.5)	52 (63.9)	34 (41.0)	75 (90.4)	83 (100)	83 (100)	80 (96.4)	82 (98.8)	82 (98.8)
**I or SDD *n* (%)**	6 (7.2)	16 (19.3)	0 (0)	0 (0)	0 (0)	0 (0)	2 (2.4)	0 (0)	1 (1.2)
**R or non-WT *n* (%)**	11 (13.3)	14 (16.9)	49 (59.0)	8 (9.6)	0 (0)	0 (0)	1 (1.2)	1 (1.2)	0 (0)

AMP, amphotericin B; ANF, anidulafungin; CAS, caspofungin; 5FC, 5-flucytosine; FLU, fluconazole; I, intermediate; ITR, itraconazole; GM, geometric mean; MCF, micafungin; POS, posaconazole; R, resistant; SDD, susceptible, dose-dependent; VOR, voriconazole.

**Table 2:** Minimum inhibition concentration values for isolates non-susceptible to one or more antifungal agents. Amino acid substitutions in the *ERG11* gene or *FKS1* gene are given where relevant.

Isolates				MIC (mg/L)						Amino Acid Substitutions ^a^
	FLU	VOR	POS	ITR	MCF	ANF	CAS	AMP	5FC	
Isolates non-susceptible to fluconazole (isolate ID no.)										
19-008-0006	>256	>8	2	>16	0.12	0.25	0.5	1	<0.06	S154F, Y132F
19-008-0016	>256	8	1	4	0.03	0.03	0.03	1	<0.06	S154F, Y132F
19-008-0007	256	>8	1	1	0.03	0.12	0.06	1	<0.06	S154F, Y132F
19-008-0008	256	>8	1	1	0.03	0.06	0.06	1	<0.06	S154F, Y132F
19-008-0017	256	8	1	1	0.03	0.03	0.03	1	0.12	S154F, Y132F
19-5326-037-0003	256	>8	0.5	1	0.03	0.06	0.25	1	<0.06	S154F, Y132F
19-008-0055	128	4	0.12	0.12	0.008	0.03	0.03	1	0.5	S154F, Y132F
19-008-0045	64	2	0.12	0.25	0.015	0.06	0.03	1	0.25	Y132F
19-008-0015	32	2	0.5	0.5	0.03	0.03	0.015	1	0.06	S154F, Y132F
19-008-0044	32	2	0.5	0.5	0.03	0.06	0.06	1	0.12	
SRR11235418 #	32	2	1	0.5	0.03	0.06	0.12	1	0.12	
19-008-0005	8	0.5	1	1	0.12	0.12	0.25	1	0.06	Y257H
19-008-0042	4	0.5	0.25	0.25	0.015	0.03	0.03	1	0.06	
19-008-0062	4	0.5	0.25	0.25	0.03	0.25	0.25	1	0.12	
19-008-0043	4	0.25	0.25	0.25	0.015	0.03	0.03	1	<0.06	
MB-21-22330	4	0.5	0.25	0.25	0.015	<0.015	0.03	1	<0.06	
19-008-0021	4	0.12	0.25	0.25	0.03	0.06	0.03	0.5	<0.06	
19-008-0063	4	0.12	0.06	0.12	0.03	0.06	0.03	0.25	<0.06	
Isolates non-susceptible to echinocandins (isolate ID no.)										
19-008-0018	2	0.25	0.5	0.25	4	0.5	4	1	<0.06	S30P
SRR11235416 #	1	0.06	0.12	0.25	2	1	8	1	<0.06	S30P

AMP, amphotericin B; ANF, anidulafungin; CAS, caspofungin; 5FC, 5-flucytosine; FLU, fluconazole; I, intermediate; ITR, itraconazole; GM, geometric mean; MCF, micafungin; POS, posaconazole; R, resistant; SDD, susceptible, dose-dependent; VOR, voriconazole; MICs given in terms of mg/L. Shading reflects classification of MIC: Red, resistant; Dark orange, non-wild type; Light orange, susceptible, dose-dependent or intermediate; Green, susceptible. Amino acid substitutions presented are recognised mutations conferring resistance in the *ERG11* gene for azole non-susceptible isolates and those in the *FKS1* gene for echinocandin non-susceptible isolates. ^a^ S154F, Y132F and Y257H represent amino acid substitutions in *ERG11*/CTRG_05283; gene annotation based on *C tropicalis* reference strain MYA-3404. S30P represents an amino acid substitution found in *FKS1*/CTRG_04661; gene annotation based on *C tropicalis* reference strain MYA-3404. # from isolate sequenced and described by McTaggart et al. [29]. Bold–homozygous mutation, not bold–-heterozygous mutation.

**Table 3:** Candidate amino acid substitutions for genes associated with azole resistance (exclusive to the azole non-susceptible cohort) and frequency of heterozygous or homozygous state in azole non-susceptible isolates.

	Description	Amino Acid Substitution	Homozygous (%)	Heterozygous (%)
**ERG11**	Sterol 14-demethylase	S154F	39	6
		Y132F	44	6
		Y257H	6	
**CDR1**	ABC multidrug transporter	P418A		6
		E697G		11
		P706T		6
**CDR11**	ABC multidrug transporter	K1467R		11
		D779Y	6	
		T332I		6
		N240K		39
		A168G		11
**MDR1**	MFS multidrug efflux pump	V15F		6
		E350K		11
		K523E		6
**MMR1**	Regulator of MDR1 expression	G697D		6
		L854S		6
**NDT80**	Activator of CDR1	A89T		6
**SNQ2**	ABC multidrug transporter	G186D		6
		V291A		6
		V629I		6
		I765V		6
		G791V		6
**STB5**	Regulator of ABC transporter expression	L129F	6	33
		T313N	6	22
**TAC1**	Inducer of ABC transporter expression	F936V		6
		A446E		33
**TPO3**	MFS multidrug efflux pump	T50I		6
**UPC2**	Regulator of ERG11	L168P		28
**YOR**	ABC multidrug transporter	T8I		6
		P44L		6
		V575A		6
		I1314T		6

Frequency in FNS isolates (*n* = 18). Highlighted are those described in the literature as associated with azole resistance.

## Data Availability

Data may be accessed by contacting the corresponding author.

## Supplementary Materials

The following supporting information can be downloaded at: https://www.mdpi.com/article/10.3390/jof8090896/s1, Table S1: Isolate details including metadata, MIC values, whole genome sequencing coverage, diploid sequence type and clonal complex, cluster picker assignment and SRA ID; Table S2: Details for isolates with downloaded sequence data including metadata, MIC values where available and cluster picker assignment; Table S3: Genes associated with antimicrobial resistance and coordinates by reference; Table S4: Amino acid substitutions noted in genes linked to fluconazole susceptibility present in homozygous form, heterozygous form or not detected; Table S5: Amino acid substitutions noted in genes linked to echinocandin susceptibility present in homozygous form, heterozygous form or not detected; Table S6: Loss of heterozygosity (LOH) in *MMR1* and *ERG11/TAC1* where detected; Figure S1: Loss of heterozygosity (LOH) plots for each of the isolates according to chromosome mapped to Assembly2020 with SNP frequency on y axis, genes as named and blue shading represents areas of LOH.
